# TRAIL-receptor 2—a novel negative regulator of p53

**DOI:** 10.1038/s41419-021-04048-1

**Published:** 2021-07-31

**Authors:** Anna Willms, Hella Schupp, Michelle Poelker, Alshaimaa Adawy, Jan Frederik Debus, Torsten Hartwig, Tim Krichel, Jürgen Fritsch, Steven Singh, Henning Walczak, Silvia von Karstedt, Heiner Schäfer, Anna Trauzold

**Affiliations:** 1 Institute for Experimental Cancer Research, University of Kiel, Kiel, Germany; 2 Centre for Cell Death, Cancer and Inflammation, UCL Cancer Institute, University College London, London, UK; 3 Department of Infection Prevention and Infectious Diseases, University of Regensburg, Regensburg, Germany; 4 CECAD Cluster of Excellence, University of Cologne, Cologne, Germany; 5 Institute of Biochemistry I, Medical Faculty, University of Cologne, Cologne, Germany; 6 Department of Translational Genomics, Center of Integrated Oncology Cologne-Bonn, Medical Faculty, University of Cologne, Cologne, Germany; 7 Center for Molecular Medicine Cologne, Medical Faculty, University Hospital of Cologne, Cologne, Germany

**Keywords:** Tumour-suppressor proteins, Cell biology

## Abstract

TNF-related apoptosis-inducing ligand (TRAIL) receptor 2 (TRAIL-R2) can induce apoptosis in cancer cells upon crosslinking by TRAIL. However, TRAIL-R2 is highly expressed by many cancers suggesting pro-tumor functions. Indeed, TRAIL/TRAIL-R2 also activate pro-inflammatory pathways enhancing tumor cell invasion, migration, and proliferation. In addition, nuclear TRAIL-R2 (nTRAIL-R2) promotes malignancy by inhibiting miRNA let-7-maturation. Here, we show that TRAIL-R2 interacts with the tumor suppressor protein p53 in the nucleus, assigning a novel pro-tumor function to TRAIL-R2. Knockdown of TRAIL-R2 in p53 wild-type cells increases the half-life of p53 and the expression of its target genes, whereas its re-expression decreases p53 protein levels. Interestingly, TRAIL-R2 also interacts with promyelocytic leukemia protein (PML), a major regulator of p53 stability. PML-nuclear bodies are also the main sites of TRAIL-R2/p53 co-localization. Notably, knockdown or destruction of PML abolishes the TRAIL-R2-mediated regulation of p53 levels. In summary, our finding that nTRAIL-R2 facilitates p53 degradation and thereby negatively regulates p53 target gene expression provides insight into an oncogenic role of TRAIL-R2 in tumorigenesis that particularly manifests in p53 wild-type tumors.

## Introduction

Tumor necrosis factor-related apoptosis-inducing ligand (TRAIL) preferentially induces apoptosis in neoplastic cells upon binding to its receptors TRAIL-R1 and TRAIL-R2 [1, 2]. This biological principle has been adopted for the development of cancer-selective therapies. However, many tumor cells are resistant to TRAIL and moreover TRAIL-R1/R2 can activate pro-inflammatory pathways thereby promoting invasion, migration, and metastasis [3–11]. All these TRAIL-R-functions are linked to their presence at the plasma membrane. Recently, it was shown that the TRAIL-Rs can translocate from the plasma membrane to the nucleus in TRAIL-dependent manner [12]. Interestingly, TRAIL-R1 and TRAIL-R2 are commonly overexpressed in cancer cells, but are frequently detected intracellularly and high intracellular abundance, especially of TRAIL-R2, was correlated with poor patient prognosis [13]. Intracellular expression was suggested to be a mechanism of TRAIL-mediated apoptosis escape [14–17]. However, in addition intracellular localization of TRAIL-Rs has been assigned pro-tumorigenic functions. As such, it was described that nuclear TRAIL-R2 negatively regulates the maturation of miRNA let-7 via interaction with the microprocessor complex and enhances tumor cell malignancy [18]. Intriguingly, both nuclear TRAIL-R1 (nTRAIL-R1) and nTRAIL-R2 are associated with chromatin suggesting their role in regulating gene expression [12].

TRAIL-R2 is a *bona fide* transcriptional target of p53, the most important tumor suppressor protein frequently inactivated in human cancers. P53 plays a central role in coordinating cellular responses to various intrinsic and extrinsic stress factors to maintain genomic stability. Depending on the stress level, p53 induces cell survival or cell death signaling pathways leading to transient or permanent cell cycle arrest (senescence) or to cell death. P53 is an unstable protein with a short half-life [19]. At physiological conditions p53 is kept at a low steady-state level and a broad network of interacting proteins regulate its stability and activity. An important negative regulator is the E3 ubiquitin ligase murine double minute 2 (MDM2). MDM2 interacts with p53, influences its cellular distribution and initiates its proteasomal degradation via ubiquitination [20, 21]. At the same time p53 transcriptionally regulates *MDM2* representing a negative feedback loop initiated by p53. Consequently, the p53-MDM2 feedback loop keeps p53 at a low level under unstressed conditions [22]. The promyelocytic leukemia protein (PML) also plays a key role in regulating the rate of p53 protein turnover. PML modulates the p53–MDM2 interaction in the nucleus thereby reducing p53 degradation [23, 24]. Most tumors escape p53 tumor suppressor functions by developing mutations or inactivating mechanisms [25]. Nevertheless, some tumors express functional p53 and treatment with chemo- or radiotherapy in these tumors activate a p53-mediated stress response. Since TRAIL-R2 is a transcriptional target of p53 [26], anti-tumor therapy has also aimed at potentiating cell death in wild-type p53-expressing malignant cells by enhancing TRAIL-R2 expression at the plasma membrane [27–29]. While regulation of TRAIL-R2 expression by p53 has been well established, the recent discovery of pro-tumoral functions of endogenous level expression of plasma membrane TRAIL-R2 [8–11] and nTRAIL-R2 [18] prompted us to investigate a potential negative feedback regulation of p53 by TRAIL-R2. Here, we show that both proteins interact in the nucleus and that TRAIL-R2 functions as a novel negative regulator of p53.

## Results

### Nuclear TRAIL-R2 co-localizes with p53

**Fig. 1:**
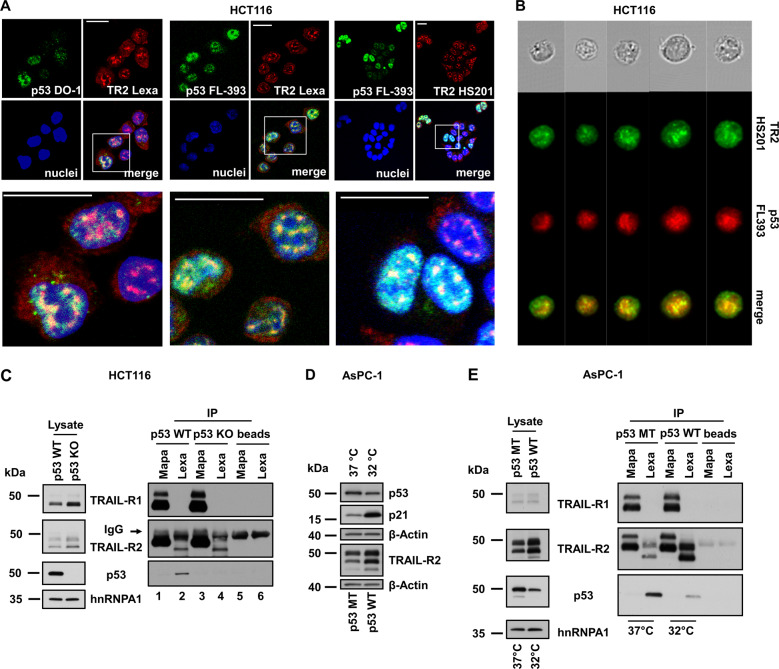
TRAIL-R2 interacts with p53 in the nucleus. Intracellular distribution of TRAIL-R2 and p53 in HCT116 cells was analyzed by indirect immunofluorescence followed by (**A**) confocal LSM and (**B**) ImageStream high-throughput microscopy. Scale bar 20 µm. The respective antibody controls are shown in Supplementary Fig. 1. **C** TRAIL-R1 and TRAIL-R2 were precipitated from nuclear fractions of HCT116 p53 WT and p53 KO cells by receptor-specific antibodies (Mapa—Mapatumumab, anti-TRAIL-R1 antibody, lane 1 and 3; Lexa—Lexatumumab, anti-TRAIL-R2 antibody, lane 2 and 4). As controls, antibodies alone were analyzed in parallel (lane 5, 6). Nuclear lysates and precipitated protein complexes were examined by western blotting (anti-p53 antibody DO-1). As gel loading control the levels of nuclear protein hnRNPA1 was analyzed in parallel. **D** AsPC-1 p53 null cells were stable transfected with a temperature-sensitive p53-mutant. These cells express mutant-p53 (p53 MT) at 37 °C and wild-type p53 (p53 WT) at 32 °C. Whole-cell lysates of AsPC-1 cells cultured for 24 h at 37 °C or 32 °C were analyzed for the presence of TRAIL-R2, p53 (anti-p53 antibody DO-1) and p21 by western blotting. The level of β-Actin was determined in parallel and served as loading control. **E** TRAIL-R1 and TRAIL-R2 were precipitated from nuclear fractions of AsPC-1 cells cultured for 24 h at 37 °C or 32 °C. As controls, antibodies alone were analyzed in parallel. Nuclear lysates and precipitated protein complexes were examined by western blotting (anti-p53 antibody DO-1). hnRNPA1 was analyzed in parallel and served as loading control.

Since both, TRAIL-R2 and p53 are present in the nucleus and each of them can interact with the chromatin and with the microprocessor complex, we asked whether both proteins may interact with each other within the nuclear compartment. First, we studied the intracellular distribution of TRAIL-R2 and p53 in wild-type p53-expressing HCT116 colon carcinoma cells by indirect immunofluorescence staining followed by confocal laser scanning microscopy (LSM). LSM analyses demonstrated co-localization of a subset of both proteins in a distinct compartment of the nucleus (Fig. 1A). ImageStream high-throughput microscopy showed also a nuclear co-localization of TRAIL-R2 and p53 in 62% of the analyzed HCT116 cells (Fig. 1B). Similar results were obtained for A549 lung cancer cells (Fig. S2).

To validate a potential interaction between TRAIL-R2 and p53, immunoprecipitation of TRAIL-R2 from nuclear fractions of wild-type p53-expressing (p53 WT) and p53 knockout (p53 KO) HCT116 cells were performed. Subsequent western blot analyses revealed the presence of p53 complexed specifically with selective TRAIL-R2-precipitates (Fig. 1C, lane 2), but not with TRAIL-R1 and -R2 containing heteromeric complexes as obtained when immunoprecipitating TRAIL-R1 using Mapatumumab (Fig. 1C, lane 1). In accordance with the absence of p53 in HCT116 p53 KO cells, no specific immunoreactivity with anti-p53 antibodies was detected in complexes with TRAIL-R2 in these cells (Fig. 1C lane 3 and 4).

To further confirm the TRAIL-R2-p53-interaction, p53 null pancreatic tumor cell line AsPC-1, retrovirally transduced with a temperature-sensitive (ts) human p53 gene was utilized [30]. Ts-p53 presents with a WT conformation at 32 °C (p53 WT) and shifts to a mutant conformation at 37 °C (p53 MT). Consistent with the WT-conformation being less stable, p53 levels decreased following a temperature switch from 37 °C to 32 °C while due to its transcriptional activity the expression of target genes such as p21 and TRAIL-R2 increased (Fig. 1D).

Immunoprecipitation of TRAIL-R2 from nuclear extracts of AsPC-1 cells clearly confirmed its interaction with p53 (Fig. 1E). The amount of p53 co-precipitated with TRAIL-R2 correlated with the overall levels of p53 and was strongly decreased at 32 °C. Of note, TRAIL-R2 interacted with p53 irrespective of its conformation required for transcriptional activity. Again, no interaction could be detected between p53 and TRAIL-R1 (Fig. 1E).

In summary, our results show a co-localization and co-precipitation of p53 with TRAIL-R2 in unstimulated cancer cells from different tumor entities.

### TRAIL-R2 affects the transcriptional output of p53

**Fig. 2:**
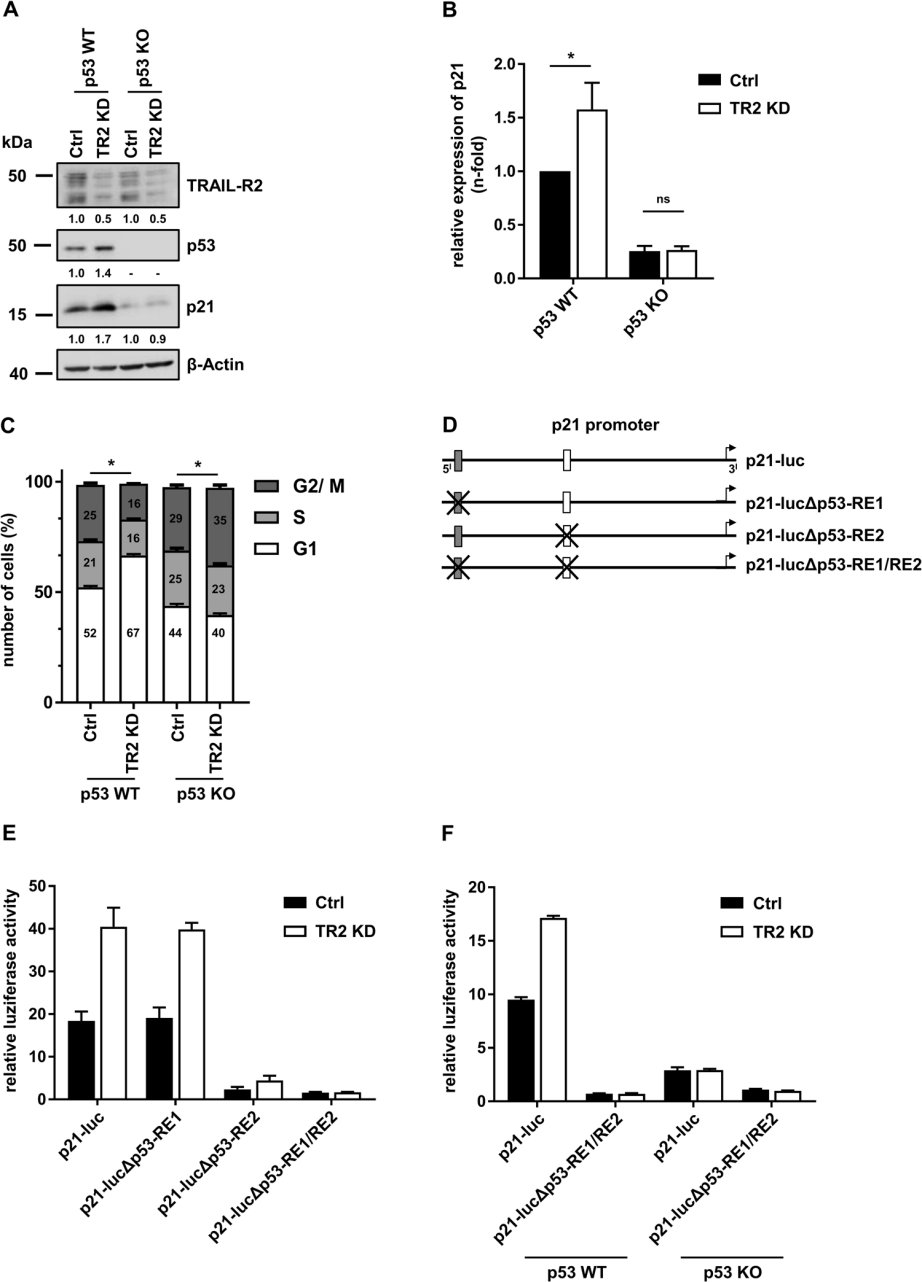
TRAIL-R2 affects the p53-mediated transcriptional regulation of the p21 gene (CDKN1A). **A** HCT116 p53 WT and p53 KO cells were transiently transfected with TRAIL-R2 siRNA (TR2 KD) or control siRNA (Ctrl). After 48 h, protein levels of TRAIL-R2, p53, and p21 were analyzed by western blotting. The level of β-Actin was determined in parallel and served as loading control. Bands were analyzed by densitometry. Intensity of each band was normalized to the corresponding β-Actin. **B** Relative expression of p21 mRNA levels (normalized to TBP) were analyzed by qRT-PCR in HCT116 p53 WT and p53 KO cells, which were transiently transfected as in (**A**) for 72 h. Bar chart shows mean values ± SD of three biological replicates (*n* = 3). **C** HCT116 p53 WT and p53 KO cells were transfected as in (**A**) and cell cycle analyses were performed 72 h later. Bar chart shows mean values ± SD of three biological replicates (*n* = 3). **D** Schematic representation of reporter vectors used in (**E**) and (**F**). **E**, **F** HCT116 p53 WT (**E**, **F**) and HCT116 p53 KO cells (**F**) were transfected with siRNA as in (**A**). After 48 h, cells were additionally transfected in duplicates with plasmids containing a luciferase gene (luc) under control of the p21-promotor (with or without p53-responsive element (RE1 or 2). After 24 h luciferase activity was measured and normalized to the activity of renilla luciferase. Bar chart shows mean values ± SD of one representative experiment (*n* = 1). ns, non significant; *p < 0.05.

Since we found that TRAIL-R2 interacts with p53 in the nucleus, we next studied whether TRAIL-R2 may influence transcriptional output of p53. Therefore, we knocked down the expression of TRAIL-R2 via siRNA (TR2 KD) in HCT116 p53 WT and p53 KO cells and analyzed p21 protein levels, the most prominent target of p53. Indeed, knockdown of TRAIL-R2 in HCT116 p53 WT cells resulted in strongly increased levels of p21 (Fig. 2A). Yet unexpected, this effect was accompanied by increased levels of p53. Importantly, consistent with the role of p53 as a transcriptional regulator of p21 expression, the enhanced p53 levels observed in TRAIL-R2 knockdown cells correlated with significantly increased mRNA levels of p21 (Fig. 2B). Of note, knockdown of TRAIL-R2 in HCT116 p53 KO cells neither changed mRNA nor protein levels of p21, suggesting TRAIL-R2-mediated regulation of p21 to function via p53 (Fig. 2A, B). In accordance with the role of p21 as a cell cycle inhibitor, knockdown of TRAIL-R2 in HCT116 p53 WT cells led to G1-phase arrest (Fig. 2C). This effect was not detectable in HCT116 p53 KO cells in which knockdown of TRAIL-R2 did not change the expression levels of p21.

Thus, we hypothesized that TRAIL-R2 may act as a novel negative regulator of p53 thereby influencing transcription of its target genes. To prove this hypothesis, we performed luciferase reporter assays using different constructs of the p21 promotor with and without p53 responsive element 1 (RE1) and RE2 [31] (Fig. 2D). HCT116 p53 WT cells were transfected with TRAIL-R2 siRNA for 48 h and subsequently with plasmids containing a luciferase gene under the control of the p21-promotor for additional 24 h. Knockdown of TRAIL-R2 led to the activation of the p21 promoter (Fig. 2E). This effect was maintained in cells transfected with p21-promotor constructs in which RE1 (p21-lucΔp53-RE1) was deleted, but was lost when either the second or both p53-REs were removed. Here, only very weak and most importantly, very similar promoter activity could be detected in control and TRAIL-R2-KD cells (Fig. 2E; p21-lucΔp53-RE2; p21-lucΔp53-RE). These results suggest that endogenous TRAIL-R2 negatively regulates the p53-transcriptional axis. To substantiate these findings, we compared the effects of TRAIL-R2-KD on the activity of the p21 promoter in HCT116 p53 WT and p53 KO cells (Fig. 2F). Again, knockdown of TRAIL-R2 in WT p53-expressing cells resulted in a strong enhancement of luciferase activity, which was abolished by the deletion of both p53-REs. No difference in luciferase activity could be detected between control cells and cells with TRAIL-R2-KD in p53 KO cells, regardless of the presence or absence of p53-REs in the p21-promoter construct (Fig. 2F).

**Fig. 3:**
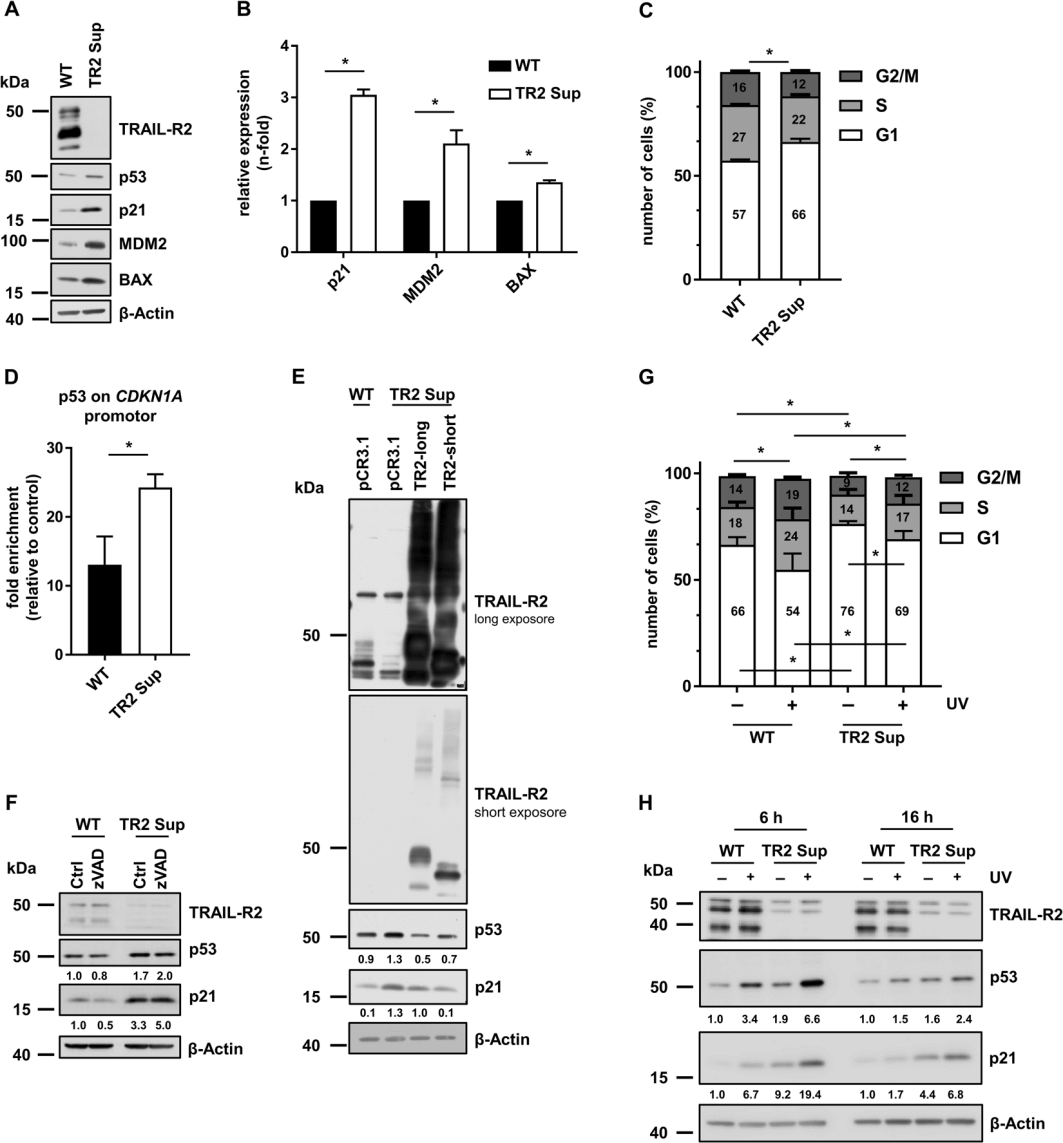
TRAIL-R2 modulates p53 transcriptional activity independently of caspases. **A** Whole-cell lysates of A549 wild type (WT) and TRAIL-R2-Sup (TR2 Sup) cells were analyzed by western blotting for the protein levels of TRAIL-R2, p53, MDM2, BAX, and p21. The level of β-Actin was determined in parallel and served as loading control. **B** mRNA levels of p21, MDM2 and BAX were analyzed by qRT-PCR in A549 cells and normalized to TBP. Bar chart shows mean values ± SD of three biological replicates (*n* = 3). **C** Cell cycle analysis through PI staining followed by flow cytometry of A549 WT and TRAIL-R2-Sup cells. Bar chart shows mean values ± SD of three biological replicates (*n* = 3). **D** Chromatin Immunoprecipitation (ChIP) was performed with anti-p53 (DO-1) and isotype control antibodies (IgG_2a_) on chromatin isolated from A549 WT and TRAIL-R2 Sup cells. DNA was extracted, and qRT-PCRs were performed using primers detecting the *CDKN1A* promotor. Enrichment was calculated as the fold increase in specific signal relative to the background signal. Results are shown ± SEM of four biological replicates (*n* = 4). **E** A549 WT and TRAIL-R2 Sup cells were transiently transfected with expression vector coding for the long (TR2-long) or short (TR2-short) isoform of TRAIL-R2, each carrying a point mutation in the death domain, or with an empty vector (pCR3.1). After 48 h, protein levels of TRAIL-R2, p53, and p21 were analyzed by western blotting. The level of β-Actin was determined in parallel and served as loading control. Bands were analyzed by densitometry. Intensity of each band was normalized to the corresponding β-Actin. **F** A549 cells were treated with zVAD-fmk (20 µM) for 48 h. Whole-cell lysates were analyzed by western blotting for the expression of TRAIL-R2, p53, and p21. The level of β-Actin was determined in parallel and served as loading control. **G** A549 WT and TRAIL-R2 Sup cells were irradiated with 10 J/m^2^ of UV-C radiation. After 16 h cell cycle analysis through PI staining followed by flow cytometry was performed. Bar chart shows mean values ± SD of three biological replicates (*n* = 3). **H** A549 WT and TRAIL-R2 Sup cells were irradiated with 10 J/m^2^ of UV-C radiation. After 6 h and 16 h whole-cell lysates were prepared and analyzed by western blotting for the expression of TRAIL-R2, p53, and p21. β-Actin was analyzed in parallel as loading control. *p < 0.05.

The TRAIL-R2-mediated regulation of p53 transcriptional output was not restricted to HCT116 cells. Suppression (Sup) of TRAIL-R2 in A549 cells also clearly increased the p53 levels. This was accompanied by enhanced expression of p53 transcriptional targets p21, Bax and MDM2, both at the mRNA and protein level (Fig. 3A, B). Again, increased levels of p21 correlated with inhibition of the cell cycle progression in the G1-phase (Fig. 3C).

To corroborate the role of TRAIL-R2 in regulating binding of p53 to the endogenous p21 promoter we performed chromatin immunoprecipitation (ChIP) assays in A549 WT and TRAIL-R2 Sup cells. Chromatin fractions from both cell lines were isolated and immunoprecipitation was performed with anti-p53 antibody or corresponding isotype control. The precipitated DNA was extracted and quantitative Real-time PCR (qRT-PCR) was performed with p21 gene (*CDKN1A)* promotor-specific primers. Importantly, qRT-PCR results show significant enrichment of *CDKN1A* promoter co-precipitated in p53-ChIP samples of TRAIL-R2 Sup cells in comparison to WT cells (Fig. 3D) substantiating the evidence that TRAIL-R2 functions as an endogenous negative regulator of p53.

Next, we tested whether the reconstitution of TRAIL-R2 in TRAIL-R2 Sup A549 cells would decrease the p53 levels. TRAIL-R2 Sup cells were transiently transfected with expression vectors encoding the long (TRAIL-R2-long) or short (TRAIL-R2-short) TRAIL-R2-isoform each carrying an inactivating point mutation in their death domain (DD). This mutation prevents the binding of FADD and consequently caspase-8 activation by clustered TRAIL death receptors and apoptosis [32]. As a control, WT and TRAIL-R2 Sup cells were transfected with an empty vector (pCR3.1). The overexpression and nuclear translocation of TRAIL-R2 isoforms were verified by western blotting and immunofluorescence analysis, respectively (Figs. 3E and S3). Consistent with the data shown in Fig. 3A, suppression of TRAIL-R2 in A549 cells led to an increased p53 level. Re-expression of either form of TRAIL-R2, diminished the p53 level, even to a lower level than that in the WT cells. Importantly, also the p21-level, which was increased in TRAIL-R2 Sup cells, was reduced in cells with reconstituted TRAIL-R2 expression (Fig. 3E). Due to mutation in the DD, the overexpressed TRAIL-R2 isoforms are not able to activate caspase-8 indicating that the observed TRAIL-R2-mediated effects on p53 levels are caspase-independent. In accordance, treatment of A549 cells with a caspase-inhibitor zVAD-fmk did not substantially changed the p53-expression levels in TRAIL-R2 Sup cells (Fig. 3F).

Next, we investigated whether TRAIL-R2 interferes with p53 induced cell cycle arrest in A549 cells after inducing DNA damage by UV-irradiation. As shown in Fig. 3G, UV-induced G1 arrest was significantly stronger in A549 TRAIL-R2 Sup cells than TRAIL-R2 WT cells. This was accompanied by much higher p53 and p21 protein levels (Fig. 3H). Interestingly, the level of p53 and p21 dropped in both cell lines over time (during 6–16 h after irradiation), but the levels of p53 and p21 in TRAIL-R2 Sup cells remained high compared with WT cells. Most importantly, and in agreement with these results, TRAIL-R2 Sup cells remained arrested in the G1 phase, whereas WT cells did not stop the cell cycle but even speeded up their proliferation (Fig. 3G).

### TRAIL-R2 regulates p53 protein stability

**Fig. 4:**
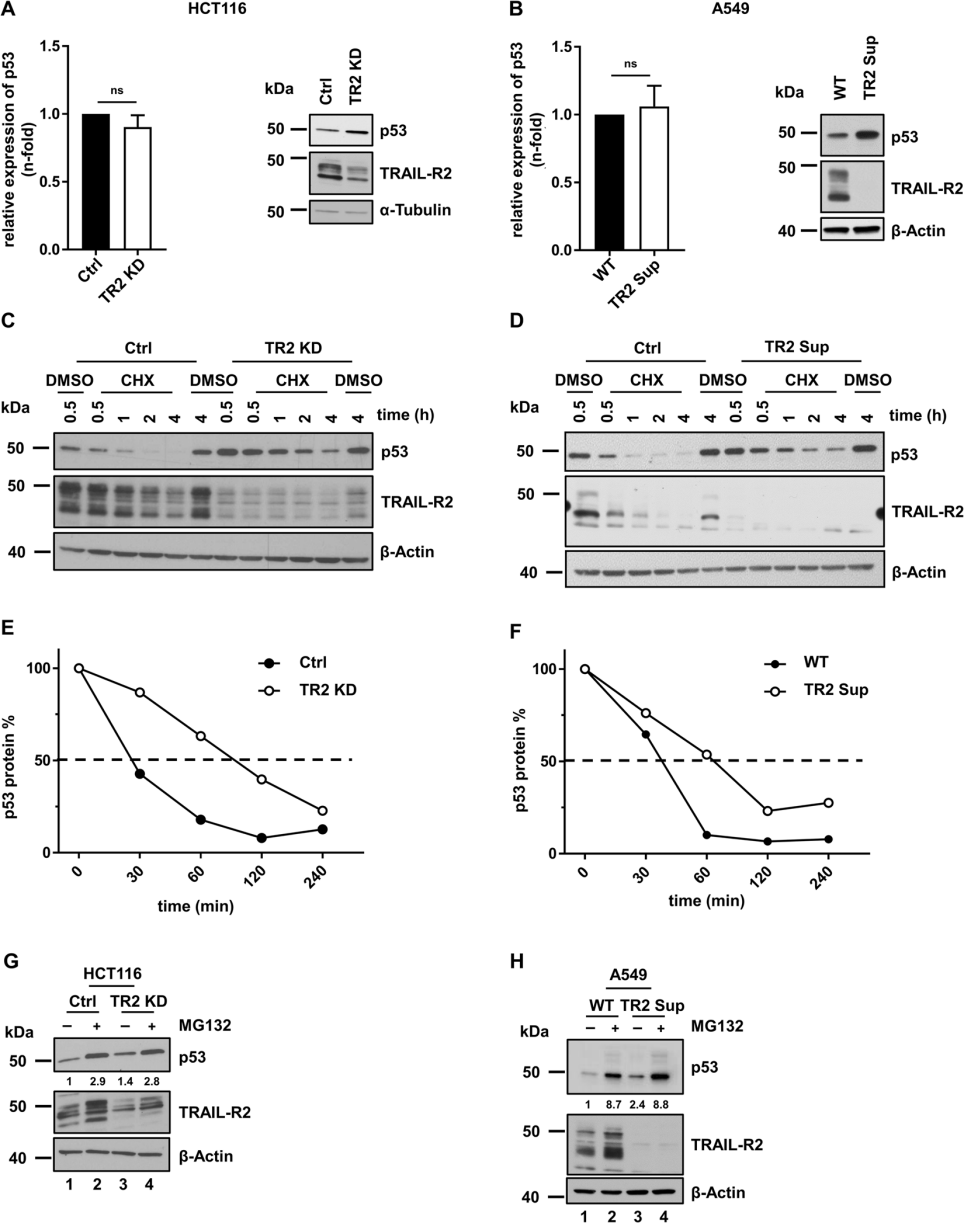
TRAIL-R2 reduces p53 half-life. **A**, **B** Relative levels of p53 mRNA (normalized to TBP) were analyzed by qRT-PCR in (**A**) HCT116 Ctrl and TRAIL-R2 KD as well as in (**B**) A549 WT and TRAIL-R2 Sup cells. Results are shown as mean of three independent experiments, each performed in triplicates ± SEM (*n* = 3). In parallel whole-cell lysates were analyzed by western blotting for the expression of TRAIL-R2 and p53. The level of β-Actin or α-Tubulin was determined in parallel and served as loading control. **C** HCT116 Ctrl and TRAIL-R2 KD or (**D**) A549 WT and TRAIL-R2 Sup cells were treated with Cycloheximide (CHX; 10 µg/ml) for indicated time periods or with DMSO as a control. Whole-cell lysates were analyzed by western blotting for the expression of TRAIL-R2 and p53. As a gel loading control, the levels of β-Actin were determined in parallel. **E**, **F** p53 protein levels were quantified by densitometry, normalized to β‐Actin and plotted against time to determine p53 half-life. **G** HCT116 Ctrl and TRAIL-R2 KD or (**H**) A549 WT and TRAIL-R2 Sup cells were treated for 4 h with MG132 (2 µM) or DMSO. Whole-cell lysates were analyzed by western blotting for the expression of TRAIL-R2 and p53. The level of β-Actin was determined in parallel and served as loading control. P53 protein level was quantified by densitometry and normalized to β‐actin. Data are shown as fold-change relative to control. ns, not significant.

To gain an insight into the mechanisms underlying the TRAIL-R2-mediated modulation of p53 levels, we asked whether TRAIL-R2 influences the p53 gene transcription. Importantly, downregulation of TRAIL-R2 expression did not change the mRNA levels of p53 neither in HCT116 nor in A549 cells, although p53 protein levels were upregulated under these conditions in both cell lines (Fig. 4A, B).

Thus, we hypothesized that TRAIL-R2 may affect the stability of p53 protein. To test this hypothesis, the half-life of p53 in HCT116 cells expressing normal and diminished TRAIL-R2 levels cultured for different time points in the presence of Cycloheximide (CHX), an inhibitor of the *de novo* protein synthesis, were compared. Western blot analyses of p53 levels revealed that knockdown of TRAIL-R2 (TR2 KD) in HCT116 cells resulted in drastically extended half-life of the p53 protein (Fig. 4C, E). This effect was even more apparent in TRAIL-R2 Sup A549 cells (Fig. 4D, F). To study the mechanisms behind this phenomenon, A549 and HCT116 cells were treated with the 26 S proteasome inhibitor MG132 and protein levels were analyzed by western blot (Fig. 4G, H). MG132 led to the accumulation of p53 in both cell lines (Fig. 4G, H lane 2 vs. 1). As already shown, inhibition of TRAIL-R2 expression, either in HCT116 or in A549 cells, also resulted in the accumulation of p53 (Fig. 4G, H lane 3 vs. 1). Importantly, concomitant inhibition of the proteasome in these cells resulted in p53 levels which were similar to that in MG132-treated control cells (Fig. 4G and H lane 4 vs. 2).

Summing up, these data show that TRAIL-R2 negatively regulates the stability of p53 protein by promoting its proteasomal degradation.

**Fig. 5:**
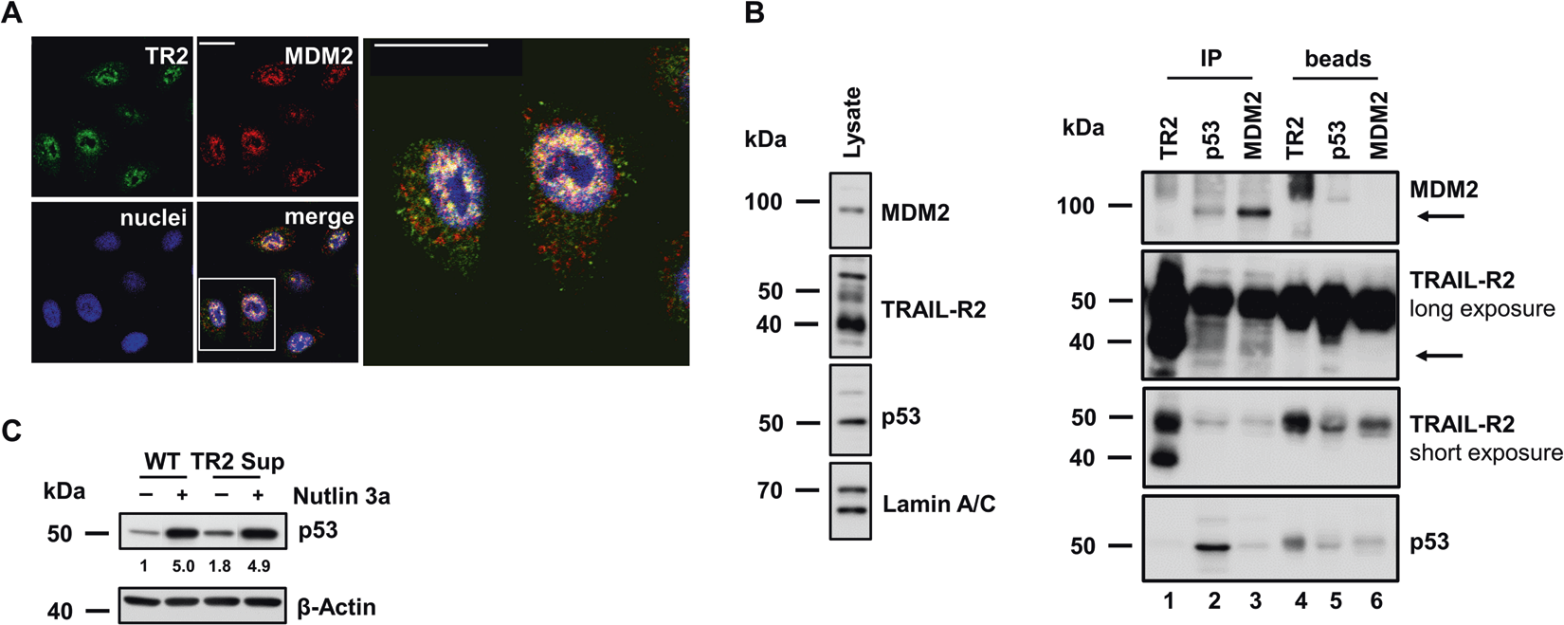
TRAIL-R2 co-localizes and co-precipitates with MDM2. **A** Intracellular distribution of TRAIL-R2 and MDM2 studied in A549 cells by indirect immunofluorescence followed by confocal LSM using Lexatumumab (anti-TRAIL-R2, Lexa) and sc-965 (anti-MDM2). Scale bar 20 µm. **B** TRAIL-R2, p53 and MDM2 were immunoprecipitated from nuclear fractions of A549 WT cells by specific antibodies [anti-TRAIL-R2 antibody (HS201); anti-p53 antibody (DO-1); anti-MDM2 antibody (sc-965)]. Nuclear lysates and precipitated protein complexes were examined by western blotting using following antibodies: anti-TRAIL-R2 antibody (2019); anti-p53 antibody (FL393) and anti-MDM2 antibody (86934). Lamin A/C was analyzed in parallel as a marker for the nuclear fraction. **C** A549 WT and TRAIL-R2 Sup cells were treated with Nutlin 3a (5 µM) for 24 h. Whole cell lysates were analyzed by western blotting for the expression of p53 using DO-1 antibody. The level of β-Actin was determined in parallel as loading control. P53 protein level was quantified by densitometry and normalized to respective β‐Actin levels. Data are presented as fold-changes relative to WT control.

Next, we asked whether MDM2 could be involved in the TRAIL-R2-mediated destabilization of p53 protein. Analyses of the intracellular distribution of both proteins by indirect immunofluorescence with confocal LSM evaluation revealed their co-localization in the nucleus (Fig. 5A). In addition, immunoprecipitation experiments performed on nuclear cell extracts confirmed the presence of TRAIL-R2 and MDM2 in the same protein complexes (Fig. 5B). In agreement with the important role of MDM2 in the downregulation of p53, disrupting the interaction of MDM2 with p53 by Nutlin 3a strongly upregulated p53 levels (Fig. 5C). This effect was clearly visible in cells, irrespective whether they expressed TRAIL-R2 (WT cells) or not (TR2 Sup cells). Importantly, Nutlin 3a-treated WT cells showed a p53 level similar to respective TRAIL-R2 Sup cells. This result suggests the crucial role of MDM2 in TRAIL-R2-mediated p53 destabilization.

### TRAIL-R2 and p53 co-localize to the promyelocytic leukemia (PML) protein

**Fig. 6:**
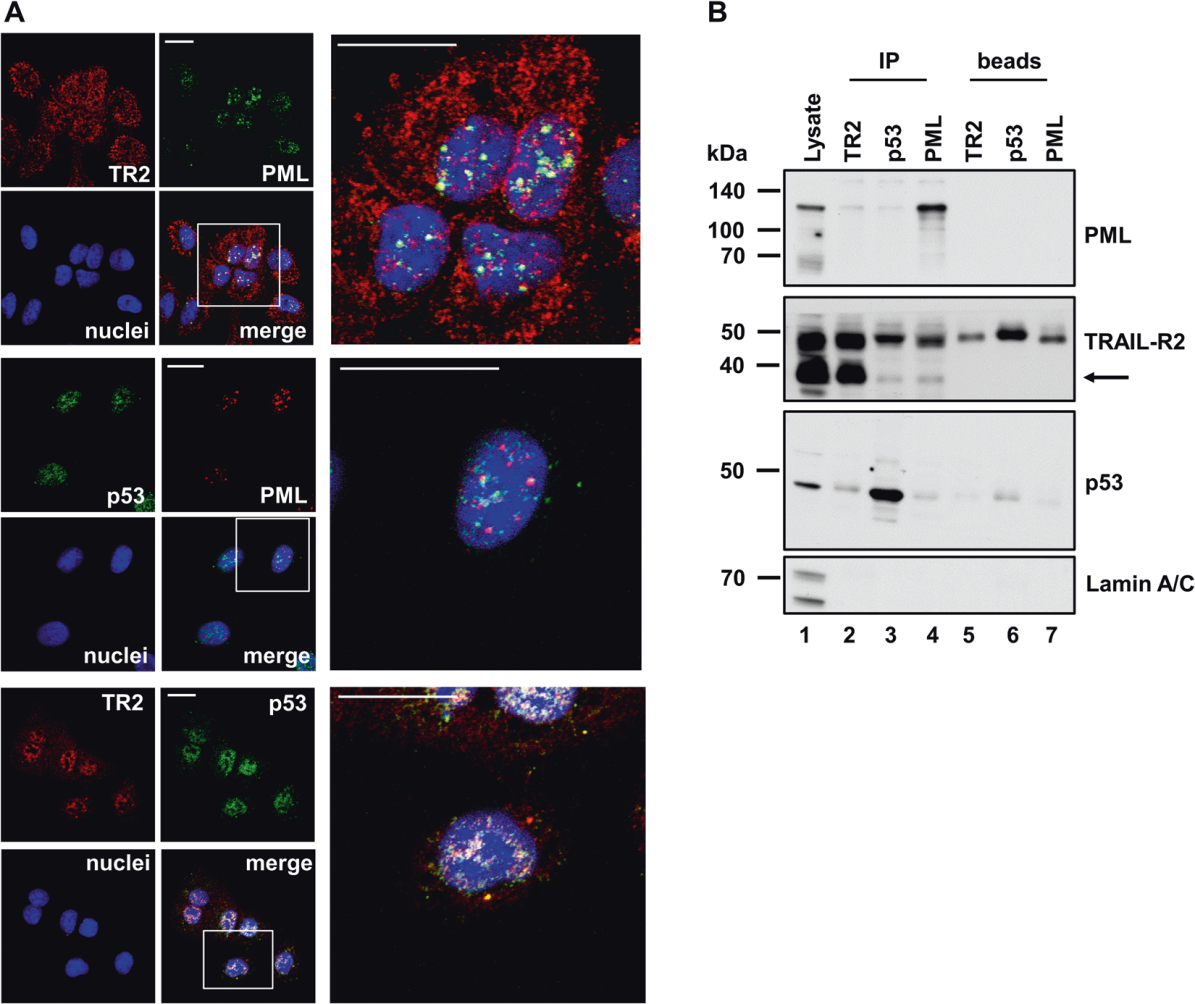
TRAIL-R2 co-localizes and co-precipitates with PML. **A** Intracellular localization of TRAIL-R2 (Lexatumumab) and PML(PG-M3), p53 (FL393) and PML (PG-M3) or TRAIL-R2 (HS201) and p53 (FL393) was analyzed by indirect immunofluorescence followed by confocal LSM in A549 WT cells. Scale bar 20 µm. **B** TRAIL-R2, p53 and PML were precipitated from nuclear fractions of A549 WT cells by specific antibodies [anti-TRAIL-R2 antibody (HS201), lane 2; anti-p53 antibody (DO-1), lane 3 and anti-PML antibody (PG-M3), lane 4]. As controls, beads with antibodies were analyzed in parallel (lane 5, 6, and 7). Nuclear lysates and precipitated protein complexes were examined by western blotting [anti-TRAIL-R2 antibody (2019); anti-p53 antibody (FL393) and anti-PML antibody (33156). Lamin A/C was analyzed in parallel as a marker for the nuclear fraction.

Given the pivotal role of PML in controlling p53 activity [33–35] and based on the staining pattern of p53 and nTRAIL-R2 revealing their co-localization in distinct sub-nuclear regions (see Figs. 1A, B and S2), immunostainings of A549 cells for PML, TRAIL-R2 and p53 were performed. Similar to p53 and TRAIL-R2, PML was localized in distinct nuclear dots, and in many of these dots its co-localization with TRAIL-R2 as well as with p53 was detectable (Fig. 6A). In addition, immunoprecipitation experiments revealed the presence of PML protein in complexes with both p53 and TRAIL-R2 (Fig. 6B).

**Fig. 7:**
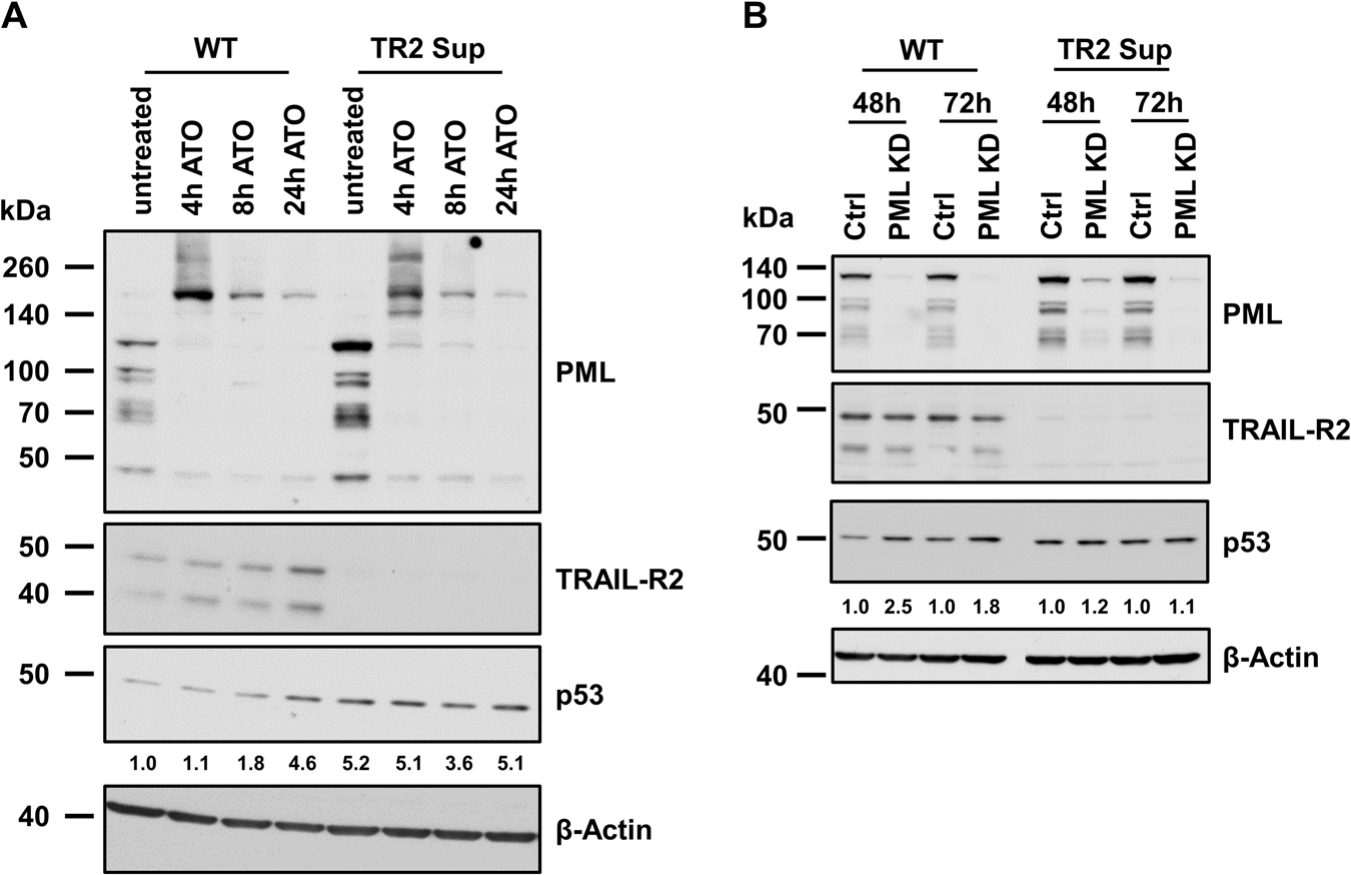
TRAIL-R2-mediated decrease of p53 stability depends on PML. **A** A549 WT and TRAIL-R2 Sup cells were treated with Arsenic trioxide (ATO; 5 µM) for indicated time periods. The expression of PML, TRAIL-R2 and p53 was analyzed in whole cell lysates by western blotting. **B** A549 WT and TRAIL-R2 Sup cells were transiently transfected with PML siRNA (PML KD) or control siRNA (Ctrl). Protein levels of PML, TRAIL-R2, and p53 were analyzed by western blotting 48 h and 72 h after transfection. The level of β-Actin was determined in parallel and served as loading control. P53 protein level was quantified by densitometry and normalized to β‐Actin.

Next, we asked whether disruption of PML-nuclear bodies (NBs) could affect the impact of TRAIL-R2 on p53 protein level. A549 cells were treated with arsenic trioxide (ATO), an agent that leads to the oxidation of PML followed by its oligomerization, polyubiquitination and subsequent degradation [36, 37]. Within 4 h of ATO treatment, oligomers and polyubiquitination products of PML at molecular weights higher than 170 kDa were formed and degraded between 8 h and 24 h of ATO treatment (Fig. 7A). Interestingly, the levels of the PML protein in all its variants between 70 and 110 kDa [38] were lower in TRAIL-R2-expressing A549 cells when compared to TRAIL-R2-deficient A549 cells. After 4 h treatment with ATO a slightly different band pattern was seen and a portion of the 110 kDa PML protein [38] was more resistant to ATO treatment until 24 h. Importantly, in control cells the amount of p53 protein as well as the expression of TRAIL-R2 increased along with the loss of PML during ATO treatment. Such effect of ATO on p53 was not visible in TRAIL-R2-deficient A549 cells. Thus, under conditions of PML-NB destruction, the expression level of TRAIL-R2 and p53 are not reciprocal as seen under regular conditions (see above), indicating that p53 degradation is not forced by TRAIL-R2 anymore. To confirm the role of PML in the TRAIL-R2-driven p53 degradation, its expression in A549 cells was abolished by siRNA (Fig. 7B). Along with the considerable loss of PML after siRNA treatment (PML KD; 48 h and 72 h), the protein level of p53 in TRAIL-R2-expressing control cells was elevated. By contrast, in TRAIL-R2-deficient cells, the higher level of p53 observed in untreated cells was not affected by the knockdown of PML indicating again that the effect of TRAIL-R2 on the stability of p53 depends on PML.

Therefore, TRAIL-R2 likely modulates the PML/p53 axis to facilitate inhibition of p53 in cancer cells.

## Discussion

Recently, it has been realized that the intracellular presence of the TRAIL death receptors is not an artifact of the histopathological staining but instead possesses a prognostic relevance for disease progression and patient’s survival [13, 18, 39]. This paradigm shift moved intracellular TRAIL receptors into the focus of current studies and resulted in the discovery of first cytoplasmic and nuclear functions of these receptors with nTRAIL-R2 being a regulator of miRNA maturation [18, 40–42]. The subsequently reported presence of TRAIL death receptors in the chromatin fraction, signalized that this is just the beginning of the story about the nuclear functions of these proteins [12]. In agreement, here we propose that nTRAIL-R2 acts as an inhibitor of the tumor suppressor protein p53. We show that both proteins interact in the nucleus and TRAIL-R2 impacts on p53 stability thereby inhibiting its transcriptional output. This represents a novel pro-tumoral function of nTRAIL-R2.

P53 orchestrates the cellular response to stress by regulating the expression of genes crucial for restoring the homeostasis or for cell death, ensuring the maintenance of the genomic stability [25]. Hence, it is not surprising that cancer cells developed mechanisms to inactivate p53 in order to escape this surveillance and to be able to accumulate mutations increasing the malignant phenotype. Approximately half of all human cancers harbor inactivating mutations in p53. The majority of remaining cancers express in fact WT p53, yet inactivated by alternative mechanisms. Thus, for cancer patients suffering from such tumors, restoring the WT p53-functions, could represent a promising therapeutic option. Overexpression of MDM2 resulting in p53-depletion represents one of the prevalent p53-inactivating mechanisms operating in cancer cells. Correspondingly, strategies aiming at the disruption of the MDM2-p53-interaction result in the stabilization of p53 and reconstitution of its tumor-suppressing functions. Such small molecules are currently tested in clinical trials for the treatment of solid tumors and hematological malignancies [43].

Recently, caspase-8, which in its cytoplasmic localization functions as a main mediator of death receptor signaling, was shown to act as a novel regulator of the p53 stability when present in the nucleus [44]. Thus, nuclear caspase-8 cleaves and thereby inactivates ubiquitin-specific peptidase 28 (USP28), the protein which stabilizes the wild-type p53 protein. This non-canonical function of caspase-8 leads to the loss of p53. Since cancer cells frequently express high levels of nuclear caspase-8, its p53-inactivating activity represents a malignancy-enhancing mechanism. Our data suggest that nTRAIL-R2-mediated regulation of p53 stability does not rely on the caspase-8 activity. Firstly, transient overexpression of TRAIL-R2 isoforms in A549 cells led to the downregulation of p53 levels despite the inability of the used TRAIL-R2-constructs to bind FADD, an adaptor protein necessary for the TRAIL receptor-mediated caspase-8 activation. Secondly, treatment of A549 cells with zVAD-fmk did not change the p53 levels in TRAIL-R2 Sup cells. Thus, the TRAIL-R2-mediated regulation of p53 stability appears to be caspase-8 independent.

Our observation that TRAIL-R2 and p53 co-localize in PML-NBs is intriguing. In these sub-nuclear structures, PML orchestrates the fate of p53 by recruiting it together with MDM2 which in turn can either ubiquitinate p53 or execute auto-ubiquitination [23, 24, 45, 46]. Under the former condition, p53 is forwarded to its proteasomal degradation, whereas it remains unaffected under the latter condition. Consequently, p53-driven cellular responses depend on its stability regulated via PML-controlled MDM2. Our data suggest that nTRAIL-R2, via its interaction with PML, interferes with this control mechanism thereby favoring MDM2-mediated p53 destabilization. Thus, PML knockdown increased p53 protein level in TRAIL-R2-expressing cells to a similar extent as the knockdown of TRAIL-R2 itself and no alteration of p53 was seen by PML knockdown in TRAIL-R2 deficient cells. This indicates that the TRAIL-R2-mediated decrease of p53 stability depends on PML. Moreover, our data show that interruption of the interaction of MDM2 and p53 by Nutlin 3a, abolishes the effect of TRAIL-R2 on p53 stability and no difference in the p53 protein level is seen anymore in TRAIL-R2 proficient and deficient cells.

Since PML directly impacts MDM2 to balance p53 ubiquitination/destabilization and MDM2 auto-ubiquitination [23, 24, 45, 46], one can speculate that TRAIL-R2, when bound to PML, prevents MDM2 auto-ubiquitination and consequently favors the ubiquitination and degradation of p53. In this fashion, TRAIL-R2 exerts its negative control of p53 along with alterations in the cell growth, e.g. through p21. This condition is an essential part of the tumor-promoting effect of TRAIL-R2 in particular when localized in the nucleus and impacting on PML-nuclear domains. Since PML bodies have a key role in the control of genomic stability by governing nuclear responses and adaptation to DNA damage or viral stress [35], enhanced nTRAIL-R2 levels in tumor cells may add to genomic instability, too. In line with this, we could demonstrate that UV-induced cell cycle arrest is blocked by nTRAIL-R2 along with robustly suppressed p53 and p21 expression levels. Accordingly, the lack of nTRAIL-R2 leads to G1 arrest accompanied by higher p53 and p21 expression.

The novel function of nTRAIL-R2 as a negative regulator of p53 suggests that this receptor may have huge impact on the tumor biology and also on the outcome of therapies aiming in the induction of p53 anti-tumor response. Keeping in mind that TRAIL-R2 is a target of p53, factors leading to the activation of p53 also lead to the upregulation of TRAIL-R2. This has been regarded as a desired anti-tumor, apoptosis-enhancing effect [26–29]. However, our unpublished results show that under such conditions TRAIL-R2 is strongly upregulated also in the nucleus, which can result, according to the here presented data, in the inhibition of the intended p53-mediated anti-tumor response.

Strikingly, our data obtained with AsPC-1 cells bearing ts-p53 variant, suggest that TRAIL-R2 may interact not only with the WT, but also with the mutant p53 making this observation potentially applicable to the majority of cancers. This together with the observed localization of nTRAIL-R2 in PML-NB, the structure with pivotal regulatory functions, as well as its interaction with the chromatin [12] suggest that this receptor might be involved in many crucial nuclear processes thus demanding further studies.

The loss of p53 is associated with poor prognosis and chemoresistance while gain of function mutants can promote cancer progression. As such p53 functionality can drastically affect therapy outcome. Since endogenous nTRAIL-R2 can modulate p53 signaling, further studies on how and to which extent nTRAIL-R2 affects tumor pathophysiology by affecting p53 is of high relevance for the clinic.

## Material and methods

### Cell culture and stimulation

The human colon cancer cell lines HCT116 p53 +/+ (p53 WT) and HCT116 p53 −/− (p53 KO) were kindly provided by Bert Vogelstein and described previously [47]. The human pancreatic carcinoma cell line AsPC-1 harboring temperature-sensitive human p53 was established in our laboratory as described previously [30]. A549 lung carcinoma cells were kindly provided by Henning Walczak. All cells were cultured in RPMI 1640 medium supplemented with 10% FCS, 2 mM glutamine and 1 mM sodium pyruvate (Thermo Fisher Scientific, Waltham, USA) and were mycoplasma-free as determined by Venor GeM Classic Mycoplasma Detection Kit (Minerva Biolabs GmbH, Berlin, Germany). For treatment experiments Cycloheximide (Sigma-Aldrich, St. Louis, USA), MG132 (Merck Millipore, Darmstadt, Germany), zVAD-fmk (Bachem, Bubendorf, Switzerland), Nutlin 3a (Sigma-Aldrich, St. Louis, USA) or Arsenic trioxide (Trisenox, Teva B.V., Haarlem, Netherlands) were used.

### Knockdown of gene expression

For transient knockdown of TRAIL-R2, cells were transfected with On-Targetplus® human TNFRSF10B SMARTpool siRNAs (L-004448-00, Horizon Discovery, Cambridge, UK) using Lipofectamine 2000 (Thermo Fisher Scientific). As controls, On-Targetplus® non-targeting pool (D-001810–10, Horizon Discovery) was used. For knockdown of PML cells were transiently transfected with On-Targetplus^®^ human PML siRNA SMARTpool (L-006547-00-0005, Horizon Discovery) using Lipofectamine RNAiMAX reagent (Thermo Fisher Scientific).

In order to obtain clone pools with stable knockdown of TRAIL-R2, HCT116 p53 WT cells were transduced with the GIPZ lentiviral shRNAmir vectors for TRAIL-R2 or with control non-silencing vectors (Open Biosystems, Huntsville, USA; CloneID: TRAIL-R2-shRNA-16711). Cells were selected with puromycin (0.5 µg/ml).

A549 TRAIL-R2 suppressed cells were generated via clustered regularly interspaced short palindromic repeats (CRISPR)-Cas-9 technology by targeting exon 1 of human TRAIL-R2. The single-guided RNA (sgRNA) sequence (CACCGACAGAACGCCCCGGCCGCTT) was generated using MIT’s CRISPR design tool (http://crispr.mit.edu/) and ligated into pSpCas9(BB)‐2 A‐GFP (PX458) vector kindly provided by Feng Zhang (Addgene plasmid #48138). A549 cells were seeded at 70% confluency and transfected with 1.25 µg of plasmid via Lipofectamine 2000 (Thermo Fisher Scientific) according to the manufacturer’s instructions. GFP expressing cells were sorted and expression of TRAIL-R2 was assessed by immunoblotting.

### Overexpression of TRAIL-R2

For transient overexpression, cells were transfected with pCR3.1 vector encoding TRAIL-R2 long or short isoform with a point mutation in the death domain or with a control vector using Lipofectamine 2000 (Thermo Fisher Scientific). Generation of TRAIL-R2 encoding plasmids was described previously [32].

### Immunofluorescence and imaging flow cytometry

Cells were washed with ice cold TBS on ice and fixed with ice cold 2.5% paraformaldehyde in PBS (10 min). After washing with TBS cells were incubated with methanol (−20 °C, 10 min), washed again with TBS and blocked with 0.5% BSA/TBS for 15 min at room temperature. Afterwards, cells were incubated with primary antibodies diluted in 0.5% BSA/TBS overnight (4 °C). Subsequently, cells were washed three times with TBS (5 min) and incubated with secondary fluorochrome-labeled antibodies as well as Hoechst 33342 (Sigma Aldrich) for 1 h at room temperature in the dark. After three washes with TBS and one wash with aqua dest., cells were mounted on glass slides using IS Mounting Medium (Dianova, Hamburg, Germany). Confocal LSM analysis was performed with a Zeiss LSM 510 (Carl Zeiss, Jena, Germany). One representative experiment out of at least three performed is shown.

Imaging flow cytometry was performed using an Amnis ImageStream^X^ MK2 (Luminex, Austin, USA) device. In brief, cells were detached using accutase. After fixation (2.5% paraformaldehyde in PBS for 10 min), cells were permeabilized using methanol (10 min at −20 °C), followed by blocking using 0.5% BSA/TBS for 15 min at room temperature and staining as described above. Hoechst staining of nuclei was omitted. Images were acquired using Amnis Inspire software (Ch2 for AF488 and Ch4 for AF546) at 60x magnification and analyzed using the co-localization wizard of the Amnis IDEAS software. One representative experiment out of at least three performed is shown.

Primary antibodies were purchased from Santa Cruz Biotechnology, Heidelberg, Germany [anti-p53 (DO-1); anti-p53 (FL393); anti-PML (PG-M3); anti-MDM2 (sc-965)], AdipoGen Life Sciences, San Diego, USA [anti-TRAIL-R2 (HS201, AG-20B-0023)] and Human Genome Sciences, Rockville, USA [anti-TRAIL-R2 (Lexatumumab)]. Fluorochrome-labeled secondary antibodies [anti-rabbit Alexa-Fluor 488 (A11008); anti-mouse Alexa-Fluor 546 (A11003); anti-mouse Alexa-Fluor 488 (A11001); anti-human Alexa-Fluor 546 (A21089)] were purchased from Life Technologies, California, USA.

### Cell fractionation and immunoprecipitation

Cells were washed with ice cold PBS, lysed with hypotonic buffer (10 mM HEPES pH 7, 10 mM KCL, 0.2 mM EDTA, 1 mM DTT) and centrifuged at 15.700 rcf (6 min, 4 °C). Supernatant represents the cytosolic fraction. Pellet was washed three times with hypotonic buffer followed each time by centrifugation at 15.700 rcf (6 min, 4 °C) and subsequently lysed in lysis puffer composed of 30 mM Tris-HCl pH 7.4, 120 mM NaCl, 1% Glycerol, 0.5% NP-40. After centrifugation at 15.700 rcf (30 min, 4 °C) supernatant containing the nuclear fraction was collected and 1 mg of protein was used for immunoprecipitation with 5–10 µg of antibody overnight. For IP presented in Fig. 1, Mapatumumab und Lexatumumab (both from Human Genome Science) were used. For IPs in Figs. 5, 6 anti-TRAIL-R2 (HS201 AG-20B-0023, AdipoGen Life Sciences), anti-p53 (DO-1), anti-PML (PG-M3) and anti-MDM2 (sc-965), all from Santa Cruz Biotechnology were used. Next day samples were incubated for 3 h with Protein G Sepharose 4 Fast Flow (GE Healthcare, Chicago, USA) or for 1 h with Pierce™ Protein A/G Magnetic Beads (Thermo Fisher Scientific). Beads were washed three times and proteins were eluted with Laemmli buffer.

### Western blot analysis

Western blot analysis was performed as described previously [48]. For whole-cell lysates, cells were lysed in RIPA buffer supplemented with protease and phosphatase inhibitors (both from Roche, Mannheim, Germany). One representative experiment out of at least three performed is shown. Primary antibodies were purchased from Cell Signaling, Frankfurt, Germany [anti-MDM2 (86934; used for all western blots); anti-p21 (2947); anti-PML (33156; used for all western blots)], Santa Cruz Biotechnology [anti-p53 (DO-1, used for all western blots except for blots shown in Figs. 5B, 6B); anti-p53 (FL393, for Figs. 5B, 6B), BD Transduction Laboratories, Heidelberg, Germany [anti-Bax (610982)], Merck Millipore, Darmstadt, Germany [anti-DR4 (AB16955)], Epitomics, California, USA [α-Tubulin (1878-1)], ProScience Incorporated, Poway, USA [anti-TRAIL-R2 (2019)] and from Sigma-Aldrich [anti-β-Actin (A5441)]. Secondary antibodies were purchased from Cell Signaling [anti-rabbit-HRP (7074) and anti-mouse-HRP (7076)].

### Dual-reporter gene assay

Dual-reporter gene assays were performed using the Dual-Luciferase® Reporter Assay System (E1910, Promega GmbH, Madison, USA). Therefore, HCT116 p53 WT and p53 KO cells were transfected with TRAIL-R2 specific siRNA or control siRNA. After 48 h cells were transfected with 1 µg plasmid coding for the firefly luciferase gene under control of the −2.2 kb human p21 promoter and 0.5 µg plasmid containing the renilla luciferase [31] for transfection efficiency control using Lipofectamine 2000 (Thermo Fisher Scientific). 24 h after plasmid transfection cells were lysed and further analyzed according to the manufacturer’s instructions. Plasmids were kindly provided by Ralf Schwanbeck (formerly Institute of Biochemistry, Kiel, Germany).

### Quantitative real-time polymerase chain reaction (qRT-PCR)

Cells were harvested, homogenized with QIAshredder (Qiagen, Hilden, Germany) and total RNA was isolated with the RNeasy Plus Mini Kit (Qiagen). Complementary DNA was synthesized using the Maxima First Strand cDNA Synthesis Kit (Thermo Fisher Scientific). The expression of p53, p21, BAX and MDM2 were studied by RT-PCR using TaqMan assays and a 7900HT Fast RT-PCR system (all from Thermo Fisher Scientific). The expression levels were calculated relative to the expression of the housekeeping gene TATA-binding protein (TBP) by the ΔΔCT method. The following assays were used: TBP (Hs00427620_m1); p53 (Hs01034249_m1); CDKN1A (Hs00355782_m1); BAX (Hs00180269_m1); and MDM2 (Hs01066930_m1).

### Propidium iodide staining

After trypsinization, cells were washed twice in cold PBS containing 5 mM EDTA (PBSE) and then resuspended in 500 µl PBSE. For fixation, 500 µl chilled EtOH was added dropwise and the mixture was incubated at room temperature for 30 min. Fixed cells were collected by centrifugation, resuspended in 500 µl PBSE, incubated with 20 µg RNase A for 30 min at room temperature and subsequently stained with propidium iodide (PI) by adding 500 µl of a 200 mg/ml PI-stock solution. Samples were stored at 4 °C in the dark until counting using a FACS Verse cytometer (Becton Dickinson, Franklin Lakes, US).

### Chromatin immunoprecipitation

The chromatin immunoprecipitation (ChIP) assay was used to determine the recruitment of p53 proteins to the CDKN1A gene promoter. 8 ×10^6^ cells were seeded in a 15 cm cell culture dish and 24 h later proteins and DNA were crosslinked using 1% Formaldehyde solution (Sigma-Aldrich). For the following steps, the SimpleChIP® Enzymatic Chromatin IP Kit (Cell Signaling Technology) was used according to the manufacturer’s instructions. Immunoprecipitations were performed with anti-p53 antibody (DO-1) or corresponding isotype control antibody (IgG_2a_). The precipitated DNA was extracted and quantitative Real-time PCR (qRT-PCR) was performed with p21 gene (*CDKN1A)* promotor-specific primers. The amount of extracted DNA was quantified via qPCR using Maxima SYBR Green/ROX master mix and a 7900HT Fast RT-PCR system (both Thermo Fisher Scientific) according to the manufacturer’s instructions. qPCR was performed with primers that detect the CDKN1A promotor [(6449), Cell Signaling Technology]. Enrichment was determined as the fold increase in a specific signal relative to the background signal.

### Induction of DNA damage

For UV-induced DNA damage, the culture medium was replaced with PBS and cells were irradiated with 10 J/m^2^ of UV-C radiation using the *GS Gene Linker* UV chamber (BioRad, Hercules, US). After irradiation, the previously removed medium was added again and cells culture was continued for various time periods.

### Statistical analyses

qRT-PCRs (Figs. 2B, 3B, D) were performed in technical triplicates. PI cell cycle measurements (Figs. 2C, 3C, G and Supplementary Fig. 4) were each performed in technical duplicates. Data from three independent experiments were included into statistical analysis. ChiP assay (Fig. 3D) was conducted with samples from four independent experiments. Statistical analyses were performed using GraphPad Prism 7.01 (GraphPad Software, San Diego, USA). Normal distribution and equal variance of the data were assumed. For comparison of two-groups two-sided *t*-test was performed. Differences between the groups were regarded statistically significant at *p*-values < 0.05 and marked with an asterisk (*). All data were included for statistical analysis with no randomization or blinding. No data points were excluded. Data are expressed as mean ± SD or SEM as indicated in the figure legends.

## Supplementary information

Supplementary figure 1

Supplementary figure 2

Supplementary figure 3

Supplementary figure 4

Supplementary figure legends
