# Supplementary figures and images for: TRAIL-receptor 2—a novel negative regulator of p53
Source: Cell Death Dis. 2021 Jul 31;12(8):757. doi: 10.1038/s41419-021-04048-1 (PMC8325694; doi:10.1038/s41419-021-04048-1)

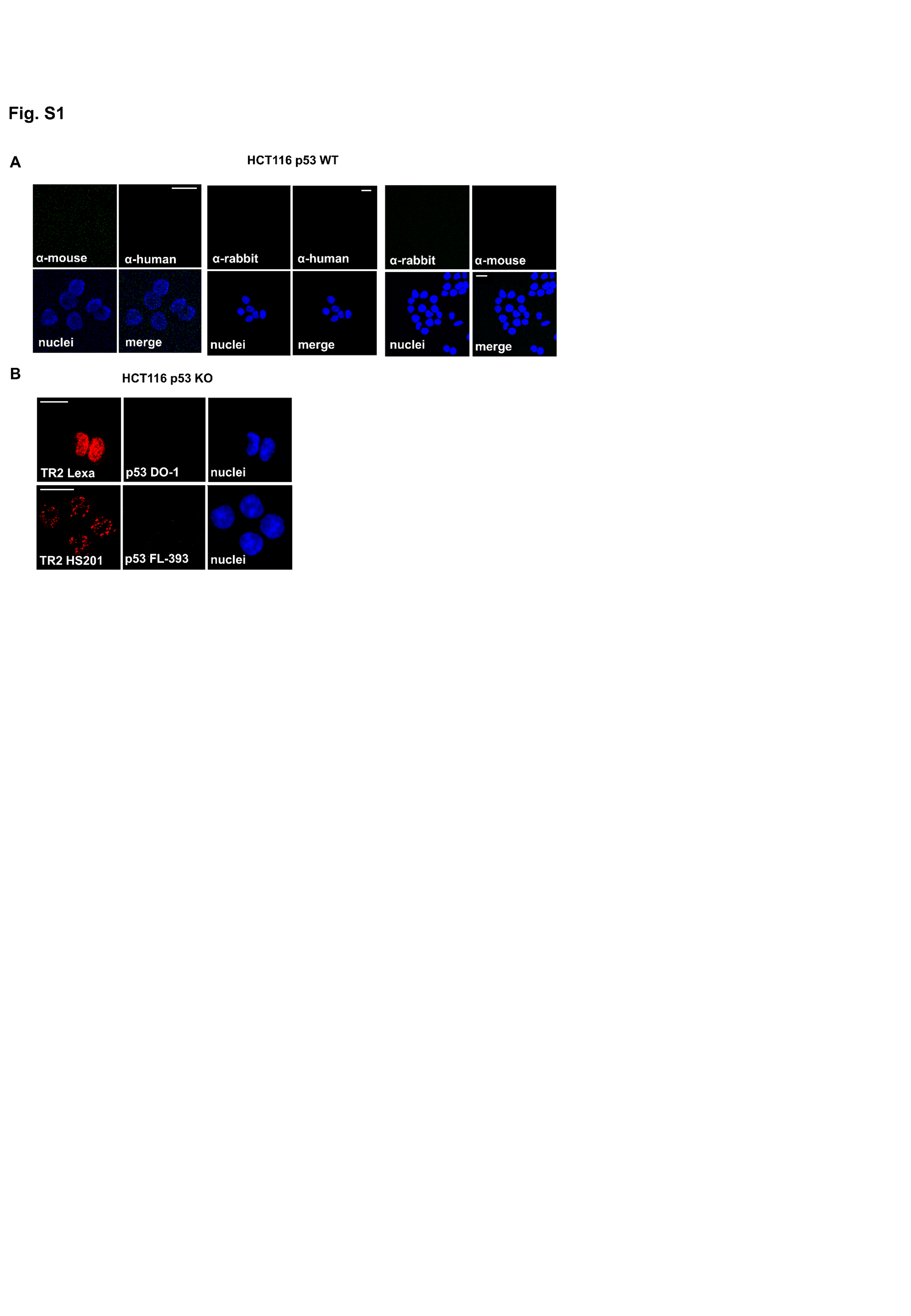

Supplement: Supplementary file 1 — Supplementary figure 1 [file 41419_2021_4048_MOESM1_ESM.tif]

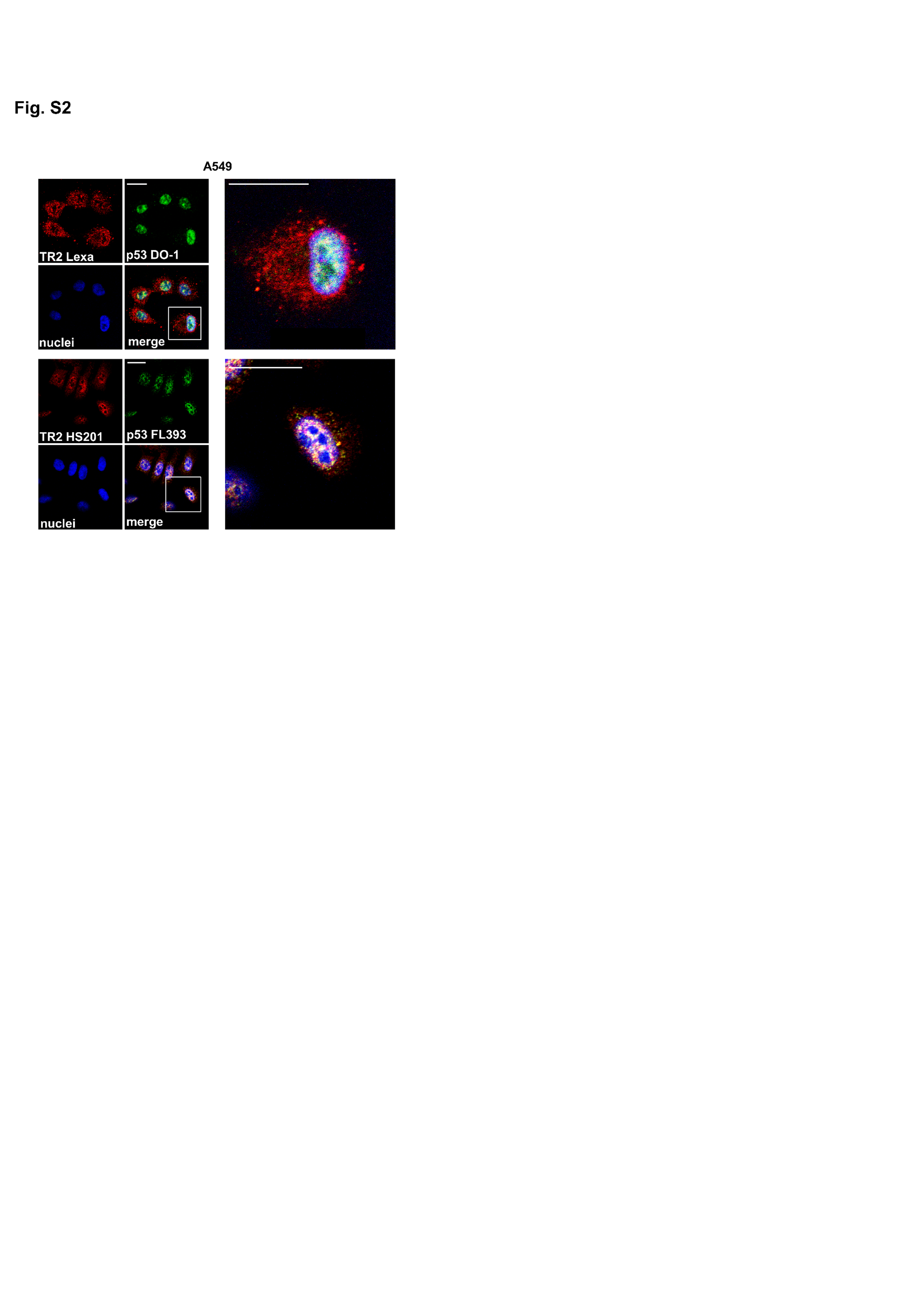

Supplement: Supplementary file 2 — Supplementary figure 2 [file 41419_2021_4048_MOESM2_ESM.tif]

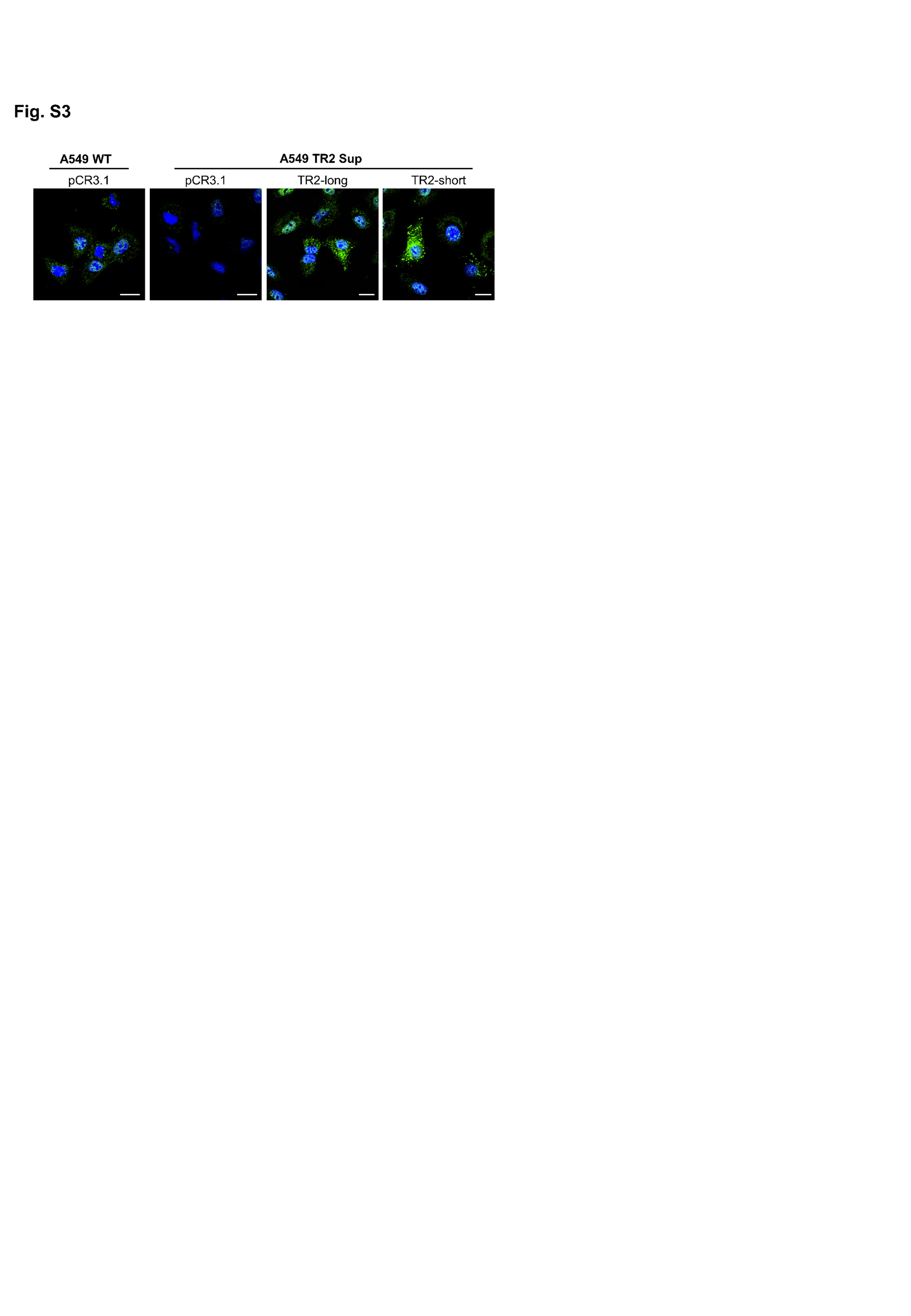

Supplement: Supplementary file 3 — Supplementary figure 3 [file 41419_2021_4048_MOESM3_ESM.tif]

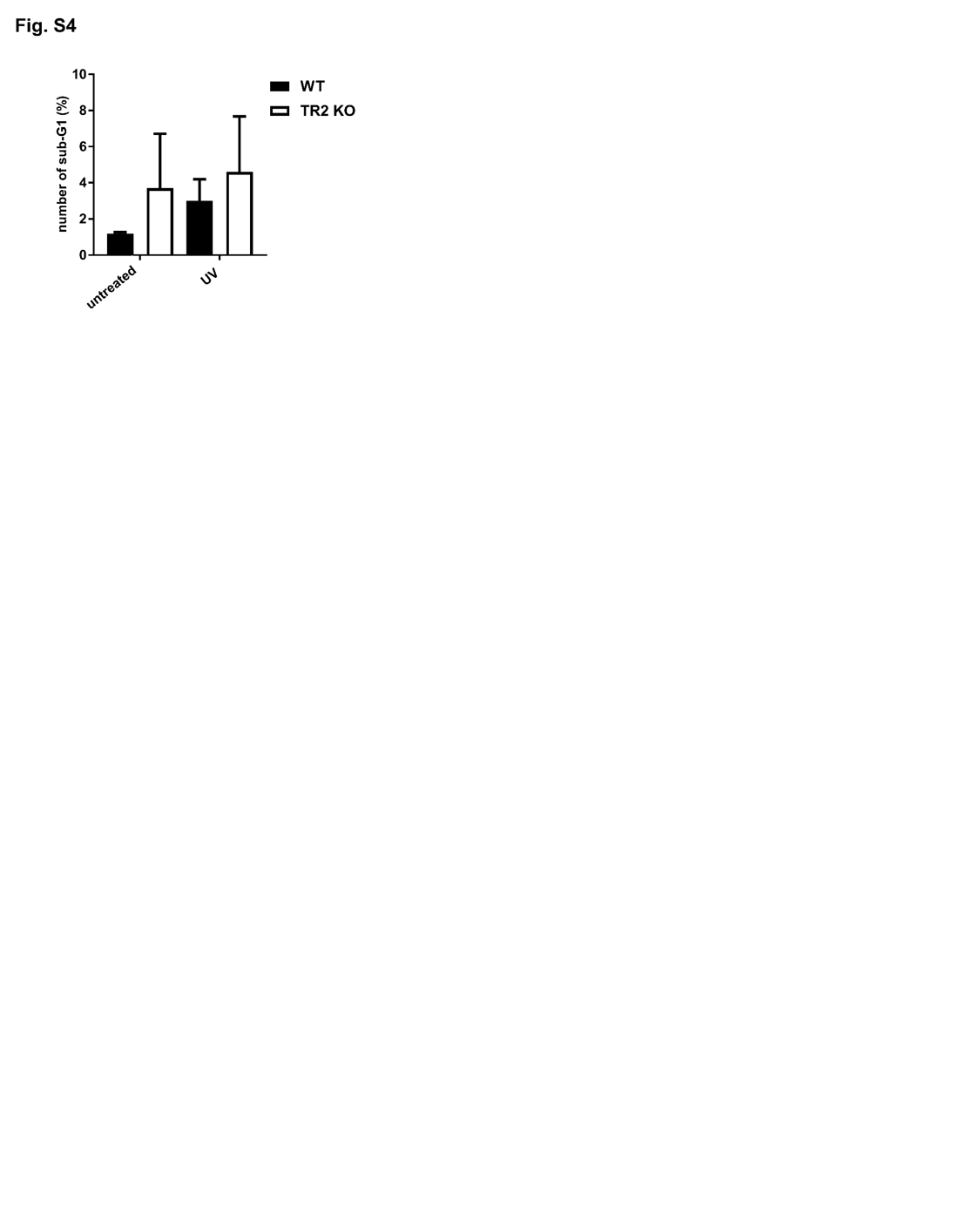

Supplement: Supplementary file 4 — Supplementary figure 4 [file 41419_2021_4048_MOESM4_ESM.tif]
