# Supplementary material for: TRAIL-receptor 2—a novel negative regulator of p53
Source: Cell Death Dis. 2021 Jul 31;12(8):757. doi: 10.1038/s41419-021-04048-1 (PMC8325694; doi:10.1038/s41419-021-04048-1)
Supplement: Supplementary file 5 — Supplementary figure legends [file 41419_2021_4048_MOESM5_ESM.docx]

**Suppl. Fig. 1: Immunofluorescence staining controls.**

**(A)** As controls for the specificity of the immunofluorescence stainings in Fig. 1A, HCT116 WT cells were incubated with the secondary antibody only and were analyzed by confocal LSM. **(B)** In addition, HCT116 p53 KO cells were stained by indirect immunofluorescence with TRAIL-R2 and p53 specific antibodies followed by confocal LSM.

**Suppl. Fig. 2: TRAIL-R2 co-localizes with p53 in A549 cells.**

Intracellular distribution of TRAIL-R2 and p53 in A549 cells was analyzed by indirect immunofluorescence followed by confocal LSM. Scale bar 20 µm.

**Suppl. Fig. 3: Intracellular distribution of transiently overexpressed TRAIL-R2 isoforms.**

A549 WT and TRAIL-R2 Sup cells were transiently transfected with expression vectors coding for the long (TR2-long) or short (TR2-short) isoforms of TRAIL-R2, both carrying a point mutation in the death domain, or with an empty vector (pCR3.1). After 24 h the intracellular distribution of TRAIL-R2 long and short isoform was studied by indirect immunofluorescence and confocal LSM.

**Suppl. Fig. 4: DNA-fragmentation of A549 cells after UV treatment.**

A549 WT and TRAIL-R2 Sup cells were irradiated with 10 J/m^2^ of UV-C radiation. After 16 h DNA-fragmentation was analyzed through PI staining followed by flow cytometry. Bar chart shows mean values ± SD of three biological replicates (n=3).
